# Integrating evidence-based lifestyle and adjunct therapies for long-term management of polycystic ovary syndrome: mechanistic insights and clinical implications

**DOI:** 10.3389/frph.2026.1821411

**Published:** 2026-05-28

**Authors:** Prasanth Babu Nandagopal, Gopika Jayakrishnan, Anu Varghese Kaithamattathil, Venkatraman Manickam

**Affiliations:** School of Biosciences and Technology, Vellore Institute of Technology (VIT), Vellore, Tamil Nadu, India

**Keywords:** dietary modification, insulin resistance, lifestyle interventions, physical activity, phytotherapy, polycystic ovary syndrome (PCOS), psychological support, traditional medicine

## Abstract

**Background:**

Polycystic ovary syndrome (PCOS) is a complex multisystem disorder affecting 6%–13% of reproductive-aged women, characterized by hormonal and metabolic dysregulation. Driven by hyperandrogenism and insulin resistance, it contributes to reproductive and cardiometabolic complications. Current management remains largely symptom-focused, often limiting long-term effectiveness and comprehensive disease control.

**Objective and rationale:**

This narrative review critically evaluates current evidence on lifestyle-based and adjunct therapeutic strategies in PCOS management, with emphasis on dietary interventions, physical activity, nutraceuticals, and traditional remedies, focusing on their potential to modulate metabolic, endocrine, and inflammatory pathways alongside conventional clinical treatments.

**Search strategy:**

A structured narrative literature search was conducted across PubMed/MEDLINE, Scopus, Web of Science, and Google Scholar to identify studies published in English-language. Keywords related to PCOS, insulin resistance, hyperandrogenism, oxidative stress, lifestyle-interventions, dietary strategies, physical activity, traditional medicine, phytotherapy and mind-body interventions were combined using Boolean operators. Relevant original studies, clinical trials, systematic reviews, and meta-analyses were selected based on scope and relevance.

**Key findings:**

Emerging evidence highlights the multifaceted role of lifestyle and adjunct interventions in improving the clinical outcomes in PCOS. Dietary approaches, including Mediterranean, low-glycemic index, ketogenic, and anti-inflammatory diets, are associated with improvements in metabolic parameters and insulin sensitivity. Structured physical activity comprising aerobic exercise, resistance training, high-intensity interval training, and mind-body practices such as yoga and Pilates demonstrates beneficial effects on metabolic and hormonal outcomes. Adjunct strategies, including nutraceuticals and selected phytochemicals, show potential in modulating insulin signalling, inflammation, and endocrine function. Psychological interventions, particularly mindfulness-based therapy and cognitive behavioural therapy, may alleviate psychosocial burden and enhance treatment adherence. Complementary cultural systems, including Ayurveda, Unani, and Traditional Chinese Medicine, have been explored as supportive approaches. Overall, heterogeneity in study design and quality necessitates cautious interpretation and further high-quality clinical validation.

**Conclusion:**

Effective PCOS management requires a balanced, evidence-based, and individualized approach in which lifestyle strategies form the foundation, pharmacological therapies remain central, and adjunct interventions are judiciously integrated as supportive measures rather than replacements for established care. Further well-designed high-quality clinical trials, standardized intervention protocols, and personalized therapeutic frameworks are needed to better establish the efficacy and safety of these strategies.

## 1 Introduction

**Figure 1:**
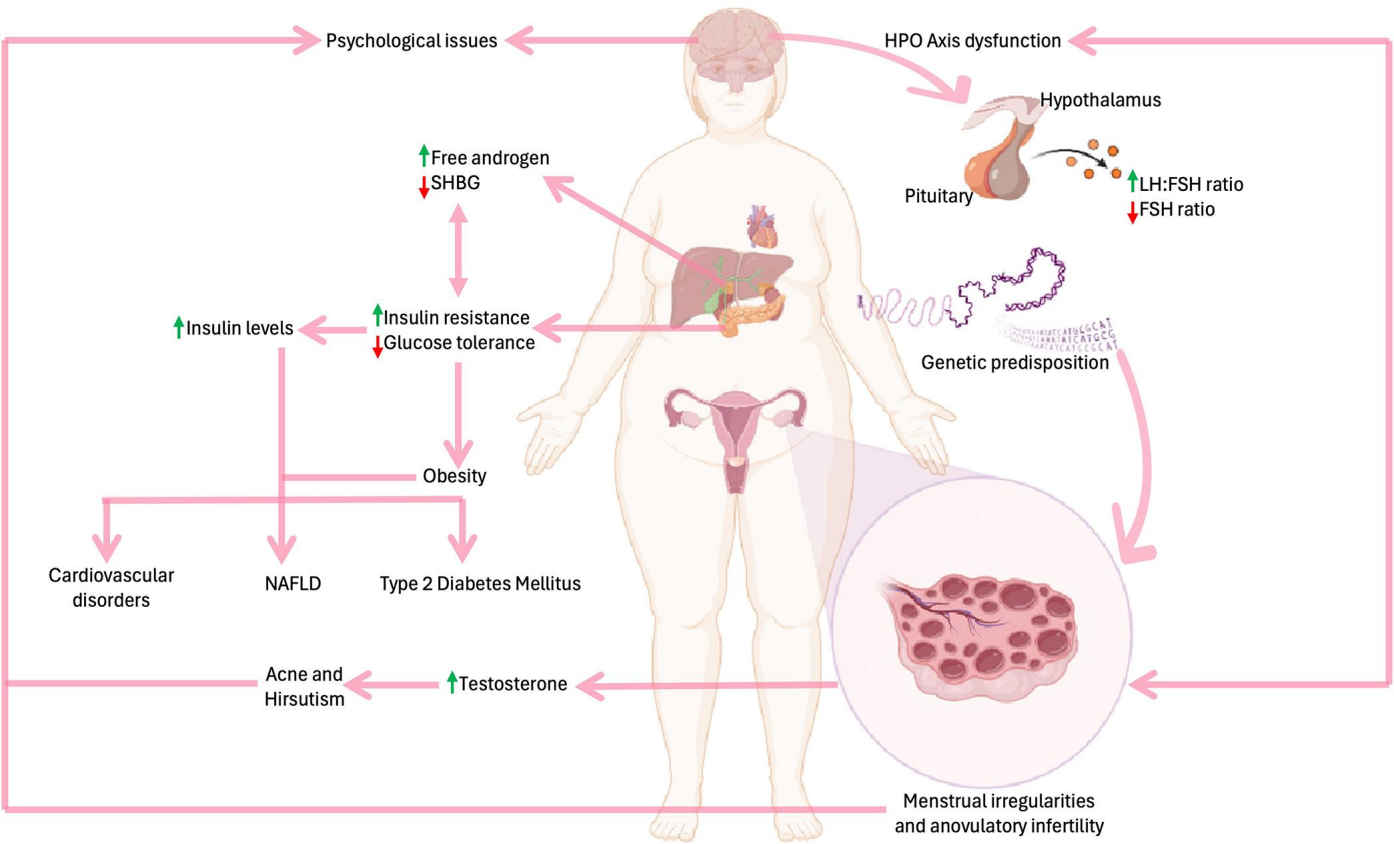
Schematic representation of the multifactorial etiology and pathophysiological mechanisms underlying polycystic ovary syndrome (PCOS). The figure illustrates the complex interplay between genetic predisposition, insulin resistance, and hyperandrogenism, accompanied by neuroendocrine dysregulation involving hypothalamic-pituitary-ovarian (HPO) axis dysfunction. Key hormonal alterations include an increased luteinizing hormone (LH) to follicle-stimulating hormone (FSH) ratio, relative reduction in FSH levels, elevated circulating insulin and glucose dysregulation, decreased sex hormone-binding globulin (SHBG), and increased free androgen and testosterone levels. These interconnected disturbances contribute to impaired folliculogenesis, anovulation, menstrual irregularities, and infertility. The figure further highlights associated clinical manifestations, including dermatological features such as acne and hirsutism, as well as broader metabolic complications, including type 2 diabetes mellitus, cardiovascular disorders, and non-alcoholic fatty liver disease (NAFLD). In addition, psychological comorbidities are represented, reflecting the neuroendocrine and psychosocial burden of the condition. Collectively, these mechanisms underscore the systemic and multifaceted nature of PCOS and its diverse reproductive, metabolic, and psychological outcomes.

Among women of reproductive age, polycystic ovary syndrome (PCOS) is one of the most prevalent endocrine disorders With an estimated global burden of 69.5 million affected individuals and increasing years lived with disability (YLDs), PCOS represents a major public health challenge. Trends over the last three decades indicate a marked rise in the prevalence (89%) and incidence (49%) of the disease within the reproductive female population with a particularly high burden reported in India (1, 2). Identifying the precise triggering factors underlying PCOS remains challenging due to the intricate interplay of multiple pathophysiological mechanisms (3), including genetic and epigenetic susceptibility, hyperandrogenism, ovarian and hypothalamic dysfunction, excessive *in utero* androgen exposure, insulin resistance, chronic low-grade inflammation, gut dysbiosis, and obesity-related metabolic processes (Figure 1) (4). Although the precise initiating mechanisms remain unclear, environmental exposures, lifestyle factors, and genetic components are believed to contribute to disease susceptibility. Several candidate gene variants, including LHCGR, FSHR, and IRF1/RAD50, have been implicated in PCOS pathogenesis (5). Clinical manifestations often begin during adolescence or early reproductive years and include irregular menstrual cycles (including heavy, prolonged, intermittent, unpredictable, or absent periods), infertility, dermatological manifestations such as acne and hirsutism (excessive facial or body hair), androgenic alopecia (male-pattern baldness or hair thinning), and metabolic features including central obesity and insulin resistance (6). These symptoms arise primarily from hormonal and metabolic dysregulation, characterized by elevated circulating androgens and alterations in reproductive hormone balance. Patients commonly exhibit increased levels of 17-Hydroxyprogesterone, dehydroepiandrosterone sulfate (DHEAS), androstenedione, anti-Müllerian hormone (AMH), and an elevated luteinizing hormone (LH) to follicle-stimulating hormone (FSH) ratio, which disrupts normal follicular development (7). As a result, follicular maturation is arrested at the small antral stage (4–8 mm), preventing the formation of a dominant follicle and contributing to chronic anovulation and infertility. Indeed, infertility affects nearly 40% of women with PCOS and accounts for approximately 90% of anovulatory infertility cases (8).

Epidemiological studies indicate that PCOS is associated with a range of adverse metabolic, reproductive, and psychological outcomes (9). Women with PCOS are at an increased risk of developing insulin resistance, abdominal obesity, dyslipidemia, cardiovascular complications, and certain malignancies, in addition to psychological conditions such as anxiety, depression, and negative body image (10). Several studies have reported that women with PCOS tend to exhibit higher body mass index (BMI) and increased visceral adiposity, even during adolescence, suggesting an early predisposition to cardiometabolic complications (7). Hyperinsulinemia is observed in a large proportion of affected individuals and plays a crucial role in disease progression by stimulating ovarian androgen production and exacerbating hyperandrogenism, which in turn contributes to the characteristic clinical manifestations of the disorder (11). Indeed, more than 80% of women presenting with hyperandrogenism are diagnosed with PCOS, and hirsutism being one of its most common clinical features, affects up to 70% of patients (12). Experimental animal studies have further suggested that prenatal exposure to elevated androgen levels may increase the susceptibility to PCOS in offspring, highlighting the potential developmental origins of the syndrome (13, 14).

Current clinical management of PCOS primarily focuses on symptom-based treatment strategies targeting hormonal imbalance, metabolic dysfunction, and reproductive complications. Pharmacological approaches commonly include combined oral contraceptives particularly those containing progestins such as norethindrone, desogestrel, or norgestimate, to regulate menstrual cycles and reduce excessive androgen. To directly combat hyperandrogenism, antiandrogen agents like spironolactone are administered. Furthermore, for patients exhibiting insulin resistance, insulin-sensitizing medications such as metformin and thiazolidinediones are prescribed to enhance metabolic function and improve insulin sensitivity. For individuals with overweight or obesity, clinical guidelines recommend lifestyle modification in combination with pharmacological therapy to reduce cardiometabolic risk. In the context of infertility associated with anovulation, letrozole is currently considered the first-line pharmacological treatment (15, 16). Despite these therapeutic options, many conventional treatments primarily address individual symptoms rather than the multifactorial pathophysiology of the disorder, highlighting the need for more comprehensive and integrative management strategies.

Current international evidence-based guidelines recommend lifestyle and weight management (17, 18), including dietary modification and regular physical activity, as the first-line strategy for the management of PCOS, with pharmacological therapies introduced based on specific clinical indications. These guidelines generally advise adherence to population-level recommendations for diet and physical activity; however, growing research interest has explored whether additional lifestyle dimensions such as sleep optimization, psychological and behavioural interventions along with selected traditional, complementary, and integrative medicine (TCIM) approaches may provide further benefits in PCOS management. Within this context, several non-pharmacological strategies have been investigated, including targeted dietary interventions that modify macronutrient composition and carbohydrate quality, structured physical activity such as aerobic exercise and yoga, and behavioural support strategies aimed at improving metabolic and psychological outcomes. In addition, a variety of complementary and integrative interventions, including nutraceuticals, phytochemicals, herbal medicines, and acupuncture, have been explored for their potential to influence key pathophysiological mechanisms underlying PCOS (19, 20), such as insulin resistance, hyperandrogenism, oxidative stress, and chronic low-grade inflammation. While conventional pharmacological therapies including hormonal contraceptives and insulin-sensitizing agents remain central to clinical management, they primarily target symptom control and may not fully address the multifactorial nature of the disorder. Consequently, there is increasing interest in multidisciplinary and integrative management strategies that combine lifestyle-based interventions with carefully evaluated adjunct therapies. Importantly, these complementary approaches should be considered supportive strategies that may complement, but not replace, guideline-directed pharmacological treatments. In this context, the present narrative review provides a comprehensive synthesis of current evidence on lifestyle interventions and adjunct therapeutic strategies for PCOS, with particular emphasis on mechanistic insights, clinical implications, and their potential integration within existing evidence-based management frameworks.

## 2 Literature search strategy

A comprehensive narrative literature search was conducted to identify relevant studies addressing lifestyle interventions and adjunct therapeutic strategies for the long-term management of PCOS. Electronic databases including PubMed/MEDLINE, Scopus, Web of Science, and Google Scholar were systematically searched for articles published in English from the earliest available records up to February 2026. The search strategy incorporated combinations of keywords and Medical Subject Headings (MeSH) such as “polycystic ovary syndrome”, “PCOS”, “lifestyle intervention”, “diet therapy”, “physical activity”, “exercise”, “nutraceuticals”, “dietary supplements”, “phytotherapy”, “complementary therapy”, “herbal medicine”, “integrative medicine”, “acupuncture”, “yoga”, “stress management”, “psychological interventions”, “traditional medicine”, “insulin resistance”, “hyperandrogenism”, “oxidative stress”, “inflammation”, and “clinical outcomes”. These terms were combined using Boolean operators (AND/OR) to refine and retrieve relevant publications. Eligible studies included original research articles, randomized controlled trials (RCTs), observational studies, systematic reviews, and meta-analyses examining lifestyle-based or adjunct therapeutic interventions relevant to PCOS management. Additional articles were identified through manual screening of reference lists from key publications. Studies not directly related to PCOS management, non-English publications, conference abstracts, and articles lacking sufficient methodological detail were excluded. The final selection of studies was guided by their relevance to the scope of this narrative review, particularly those providing insights into the mechanistic basis and clinical implications of lifestyle and adjunct therapies in PCOS management.

## 3 Pathophysiology

The pathophysiology of PCOS is multifactorial and involves interactions among hyperandrogenism, insulin resistance, neuroendocrine dysregulation, and altered adipose tissue function, which collectively contribute to disease development and progression (21, 22). Clinically, PCOS manifests through a spectrum of symptoms including hirsutism, menstrual irregularities, obesity, infertility, and metabolic disturbances. Morphologically, PCOS is associated with the presence of multiple small antral follicles due to impaired follicular maturation. Polycystic ovarian morphology is commonly defined by the presence of ≥12 follicles measuring 2–9 mm in diameter and/or increased ovarian volume (>10 mL) in at least one ovary (23).

### 3.1 Hyperandrogenism

Hyperandrogenism is a key feature of PCOS and plays a central role in both reproductive and metabolic abnormalities. Dysregulation of ovarian theca cells leads to excessive production of androgens such as testosterone and androstenedione (21). This process is often associated with altered signalling within the hypothalamic-pituitary-ovarian (HPO) axis, resulting in an increased secretion of luteinizing hormone (LH) relative to follicle-stimulating hormone (FSH) (24). Elevated LH levels stimulate ovarian androgen synthesis, further exacerbating hormonal imbalance. Excess androgen levels interfere with normal follicular development, preventing the selection of a dominant follicle and leading to chronic anovulation and menstrual irregularities. Clinically, hyperandrogenism contributes to manifestations such as hirsutism, acne, and hyperpigmentation.

### 3.2 Menstrual irregularities and infertility

Reproductive dysfunction in PCOS largely results from impaired folliculogenesis and ovulatory failure. Hormonal imbalance and metabolic disturbances disrupt the coordinated signalling within the HPO axis, resulting in irregular ovulation or anovulation (25). Elevated levels of AMH, produced by granulosa cells of developing follicles, are frequently observed in women with PCOS. Increased AMH reflects the higher number of small follicles and may further inhibit follicular maturation, contributing to infertility (26, 27). Environmental factors such as exposure to endocrine-disrupting chemicals, including bisphenol-A and phthalates, have also been implicated in disrupting metabolic and reproductive pathways associated with PCOS (28, 29).

### 3.3 Obesity and PCOS

PCOS is commonly associated with hormonal imbalance and metabolic disturbances, including obesity. Genetic susceptibility and androgen-mediated alterations in lipid metabolism contribute to insulin resistance and dyslipidemia and obstructive sleep apnoea, which are observed in many women with PCOS (24, 30). Elevated insulin levels reduce sex hormone-binding globulin (SHBG), increasing circulating androgens and further exacerbating hyperandrogenism and metabolic dysfunction (31). Additionally, visceral fat accumulation is frequently observed in PCOS, although BMI alone may not accurately reflect differences in body fat distribution (32). According to international PCOS management guidelines (17, 18), lifestyle modification remains the first-line strategy for improving metabolic and reproductive outcomes, particularly in individuals with overweight or metabolic dysfunction, as sustained weight management and targeted lifestyle interventions can significantly improve PCOS symptoms, including those related to infertility (33).

### 3.4 Hyperinsulinemia

Insulin, a key metabolic hormone secreted by pancreatic β-cells, plays a central role in regulating glucose homeostasis and energy metabolism. In PCOS, insulin resistance and compensatory hyperinsulinemia are common metabolic abnormalities that contribute to dyslipidemia and increase the risk of type 2 diabetes and cardiovascular disease (33). Elevated insulin levels also exacerbate reproductive dysfunction by reducing SHBG and stimulating ovarian androgen production, thereby increasing circulating androgen levels and contributing to menstrual irregularities and anovulation (24). Metabolic disturbances associated with hyperinsulinemia frequently coexist with adiposity and impaired glucose tolerance in women with PCOS (22). In addition to metabolic complications, PCOS is also associated with psychological comorbidities, including anxiety, depression, and eating disorders, which can negatively impact quality of life (34, 35). Given the multifactorial endocrine nature of PCOS, effective management requires integrated strategies combining lifestyle interventions with appropriate pharmacological therapies (36).

## 4 Current clinical treatment strategies for PCOS

Clinical management of PCOS primarily focuses on symptom control, prevention of long-term metabolic complications, and improvement of reproductive outcomes. Because the clinical presentation varies among patients, treatment is typically individualized and targeted toward specific manifestations such as menstrual irregularities, hyperandrogenism, infertility, and metabolic dysfunction (37). Current clinical approaches include pharmacological therapy, hormonal regulation, metabolic management, and selected adjunct therapies.

### 4.1 Pharmacological and hormonal management

Pharmacological therapy remains a cornerstone of evidence-based clinical management for PCOS and is primarily directed toward correcting hormonal imbalance, improving metabolic parameters, and restoring ovulatory function. Combined oral contraceptives (COCs) are recommended as first-line therapy for women who are not seeking pregnancy, with evidence from randomized controlled trials and clinical guidelines supporting their efficacy in regulating menstrual cycles, suppressing ovarian androgen production, and alleviating clinical manifestations of hyperandrogenism such as hirsutism and acne (38, 39). However, long-term use of COCs may be associated with adverse effects, including an increased risk of thromboembolism, potential cardiometabolic complications, altered glucose metabolism, weight gain, and mood disturbances, necessitating careful patient selection and monitoring (40). For women presenting with insulin resistance, insulin-sensitizing agents such as metformin are widely utilized and are supported by moderate-quality clinical evidence (41, 42). Metformin improves insulin sensitivity, reduces hepatic glucose production, and may contribute to modest weight reduction (43). While it has demonstrated benefits in improving metabolic parameters and, to some extent, ovulatory function, its effects on menstrual cycle regulation and fertility outcomes are less consistent compared with COCs and ovulation induction agents, and its clinical utility may be limited by gastrointestinal intolerance affecting adherence.

Anti-androgen medications are employed to address symptoms of hyperandrogenism. Agents such as spironolactone and finasteride act by antagonizing androgen receptors or inhibiting androgen synthesis, thereby reducing hirsutism and acne (41, 42). Evidence supports their effectiveness as adjunct therapies; however, their use requires careful consideration of safety profiles. Spironolactone is contraindicated during pregnancy due to potential teratogenic effects and may be associated with adverse outcomes such as menstrual irregularities, amenorrhea, and hyperkalemia, particularly in patients receiving concomitant potassium-sparing medications (44–48). Management of infertility in PCOS typically involves ovulation induction therapies, with letrozole and clomiphene citrate recommended as first-line pharmacological agents based on evidence from randomized controlled trials demonstrating improved ovulation and pregnancy rates (49, 50). In cases of treatment resistance, assisted reproductive technologies such as *in vitro* fertilization (IVF) may be considered. Gonadotropin therapy, commonly used in IVF protocols to stimulate follicular development, is effective but associated with clinically significant risks, including ovarian hyperstimulation syndrome (OHSS), multiple pregnancies, and ovarian cyst formation (51, 52). Accordingly, these interventions require individualized dosing and close monitoring of follicular and hormonal responses. Management of infertility in PCOS patients typically involves ovulation induction therapies. Clomiphene citrate and letrozole are widely used as first-line pharmacological agents to stimulate ovulation (49, 50). In cases where these treatments are unsuccessful, assisted reproductive techniques such as *in vitro* fertilization (IVF) may be considered. Gonadotropin therapy is frequently employed during IVF cycles to promote follicular development; however, these interventions carry risks such as ovarian hyperstimulation syndrome (OHSS), multiple pregnancies, and ovarian cyst formation (51, 52). Therefore, careful monitoring of follicular growth and hormonal responses is necessary during treatment. Collectively, these pharmacological interventions constitute the foundation of PCOS management.

### 4.2 Adjunctive and complementary therapeutic approaches

In addition to pharmacological management, adjunct therapies including nutritional supplementation and complementary medicine have been explored to support metabolic and reproductive health in women with PCOS. Several dietary supplements have shown potential benefits in improving metabolic parameters and hormonal balance. For instance, omega-3 fatty acids possess anti-inflammatory properties and may contribute to improved lipid profiles, reduced cardiovascular risk, and modulation of androgen levels (53, 54). Similarly, vitamins such as vitamin D and vitamin E have been investigated for their potential roles in improving insulin sensitivity, menstrual regularity, and overall metabolic health (55, 56). Inositol supplementation, particularly myo-inositol and D-chiro-inositol, has gained considerable attention in recent years. These compounds function as insulin-sensitizing agents and have demonstrated promising effects in improving ovulatory function, reducing circulating androgen levels, and correcting metabolic disturbances associated with PCOS.

Complementary therapies are also increasingly utilized alongside conventional medical treatment to enhance overall well-being. Acupuncture, including electro-acupuncture, has been reported to influence neuroendocrine regulation and improve glucose and lipid metabolism, thereby contributing to the management of symptoms such as menstrual irregularities, hirsutism, and acne (57, 58). Additionally, several herbal remedies have been investigated for their potential therapeutic benefits (59). For example, cinnamon has been reported to improve lipid metabolism and menstrual regularity (60), while *Glycyrrhiza glabra* (licorice) exhibits anti-inflammatory and antioxidant properties that may contribute to reduced testosterone levels (60). Other plant-based interventions such as *Vitex agnus-castus*, fenugreek, black cumin, and flaxseed have also been explored for their potential roles in regulating hormonal balance, improving fertility outcomes, and enhancing insulin sensitivity (61). Despite the availability of diverse therapeutic options, pharmacological treatments are often associated with adverse effects and may provide only partial symptom relief, particularly in individuals with severe metabolic dysfunction (62). Moreover, prolonged use of certain medications and high-dose supplementation may lead to unwanted side effects or toxicity (63, 64). These limitations highlight the need for integrative and patient-centered management strategies that combine evidence-based medical therapy with supportive lifestyle and complementary interventions to optimize long-term outcomes in PCOS.

## 5 Lifestyle factors in the development and management of PCOS

### 5.1 Metabolic and behavioural lifestyle determinants in PCOS

Lifestyle factors play a critical role in both the development and management of PCOS. Modifiable behaviours such as dietary habits, physical activity, stress management, and sleep patterns significantly influence metabolic, endocrine, and reproductive outcomes associated with the disorder (65). International evidence-based guidelines for PCOS management identify lifestyle modification as first-line therapy, emphasizing its central role in addressing metabolic dysfunction and improving clinical outcomes in affected individuals (17). Nutritional interventions are central to improving metabolic health in women with PCOS. A balanced dietary pattern that provides adequate macro- and micronutrients can contribute to improved metabolic regulation, hormonal balance, and overall well-being. Obesity is one of the most prevalent features associated with PCOS and is strongly linked to insulin resistance, cardiovascular disease risk, infertility, and psychological complications (66). Consequently, weight management is often considered a primary therapeutic target in PCOS and other metabolic disorders (67, 68). International guidelines, including recommendations from the World Health Organisation (WHO), emphasize maintaining a healthy body weight through appropriate energy balance between dietary intake and physical activity. These guidelines suggest limiting total dietary fat intake to less than 30% of total energy, reducing saturated fats to below 10% and trans fats to less than 1%, and restricting free sugars to under 10% of daily energy intake, preferably below 5%. Adherence to such dietary recommendations may help improve BMI, insulin sensitivity, reproductive hormone balance, and ovarian function in women with PCOS. In addition, specific dietary strategies such as low glycemic index (GI) and low glycemic load (GL) diets have been shown to reduce the risk of type 2 diabetes and cardiovascular disease. Other dietary approaches, including high-protein diets, monounsaturated fatty acid (MUFA)-enriched diets, and calorie-controlled nutritional plans, may further support metabolic health and psychological well-being in affected individuals (19). Physical activity represents another key lifestyle component influencing PCOS outcomes. A controlled clinical study demonstrated that structured exercise training significantly improves insulin sensitivity in women with PCOS by enhancing skeletal muscle glucose uptake and metabolic function (69). Aerobic exercise and progressive resistance training have been associated with improvements in metabolic parameters and may contribute to restoration of ovulatory function and menstrual regularity. Exercise interventions may also influence endocrine parameters, including circulating androgen levels and sex hormone-binding globulin (SHBG) concentrations. In addition to conventional exercise modalities, mind-body practices such as yoga have gained increasing attention for their potential benefits in PCOS. Yoga integrates physical movement, controlled breathing, and mindfulness, which may collectively support hormonal regulation and metabolic balance (22). Behavioural strategies such as mindfulness-based meditation and cognitive behavioural therapy (CBT) have also been explored as complementary approaches for managing stress and improving psychological health in women with PCOS (70).

### 5.2 Sleep and circadian rhythm dysregulation in PCOS

**Figure 2:**
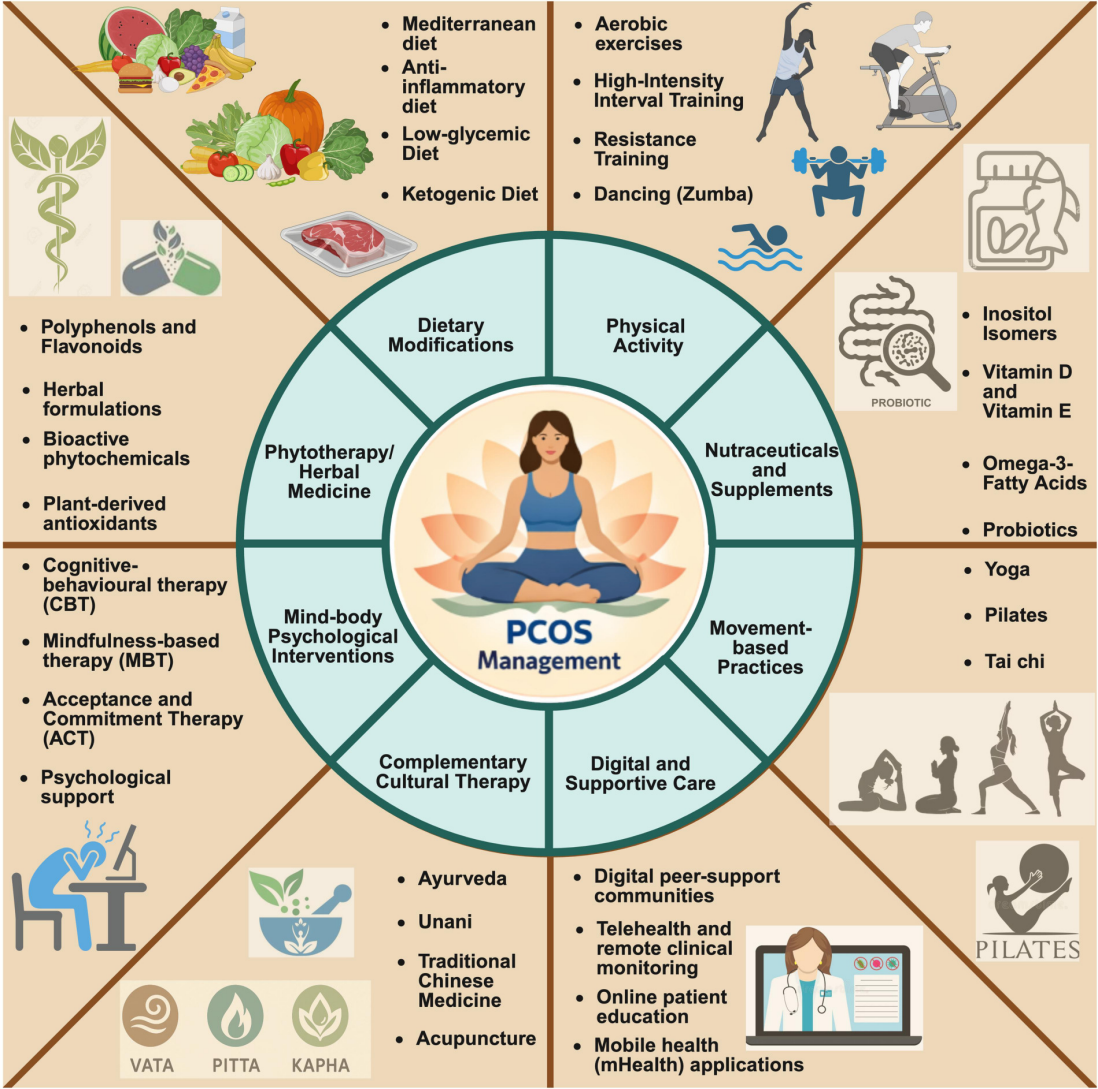
Schematic overview of evidence-informed lifestyle interventions and adjunct therapeutic strategies for the integrative management of polycystic ovary syndrome (PCOS). The figure illustrates key intervention domains, including dietary modifications (Mediterranean, low-glycaemic index, anti-inflammatory, and ketogenic dietary patterns), structured physical activity (aerobic exercise, resistance training, and high-intensity interval training), and movement-based practices (yoga, Pilates, and tai chi). Adjunct strategies encompass nutraceuticals and bioactive compounds (inositol isomers, vitamin D and E, omega-3 fatty acids, probiotics), phytotherapy and herbal medicine (polyphenols, flavonoids, herbal formulations, phytochemicals, plant-derived antioxidants), and complementary cultural therapies (ayurveda, unani, and traditional Chinese medicine, acupuncture). Psychological and mind-body interventions (mindfulness-based therapy, cognitive-behavioural therapy, acceptance-commitment therapy and stress management strategies), are also represented, alongside digital and supportive care tools (telehealth, mobile health applications, digital-peer support communities and online patient education). These interventions are depicted as complementary approaches targeting key pathophysiological features of PCOS, including insulin resistance, hormonal dysregulation, inflammation, and psychosocial burden, and are intended to function as supportive components within a patient-centred framework when integrated with conventional medical management.

Sleep quality and circadian rhythm regulation are emerging lifestyle factors that may significantly influence PCOS pathophysiology. A systematic review and meta-analysis reported that women with PCOS have a significantly higher prevalence of sleep disturbances, which are associated with adverse cardiometabolic profiles, including increased fasting glucose, dyslipidaemia, elevated blood pressure, and central adiposity (71, 72). Alterations in circadian rhythm and elevated cortisol levels may further impair insulin sensitivity and contribute to metabolic abnormalities. Inadequate sleep has also been linked to reduced melatonin production, altered appetite-regulating hormones such as leptin, and increased susceptibility to obesity and metabolic disorders (73). Moreover, women with PCOS may experience higher prevalence of sleep fragmentation, reduced rapid eye movement (REM) sleep, and prolonged sleep onset latency compared with individuals without PCOS. Obstructive sleep apnoea (OSA), characterized by recurrent upper airway obstruction and intermittent hypoxia, can further exacerbate β-cell dysfunction, glucose intolerance, and cardiovascular complications (74). Short sleep duration has also been associated with increased risk of anovulation and altered reproductive hormone levels (75). Collectively, these findings highlight the importance of comprehensive lifestyle strategies in PCOS management. Integrating dietary optimization, regular physical activity, stress reduction, and adequate sleep with conventional medical therapies may provide a holistic approach for improving metabolic, reproductive, and psychological outcomes in individuals affected by PCOS (Figure 2).

## 6 Phytotherapy for PCOS: beyond conventional approaches

Growing interest in integrative strategies for the management of PCOS has led to increasing investigation of plant-derived bioactive compounds as potential adjunct therapies. Several classes of phytochemicals have been investigated for their potential influence on metabolic and endocrine disturbances associated with PCOS (76). Compounds such as polyphenols, flavonoids, carotenoids, and dietary fibres have been reported to exert antioxidant, anti-inflammatory, and insulin-sensitizing effects that may contribute to improvements in metabolic regulation (77). Experimental and preliminary clinical studies suggest that these compounds may influence key features of the disorder, such as insulin resistance, hyperandrogenism, oxidative stress, and lipid dysregulation. Evidence from preclinical research also suggests that certain plant extracts, including *Rubia cordifolia*, may improve ovulation, follicular development, and metabolic parameters in experimental PCOS models (78, 79). Accordingly, the following sections examine therapeutic herbs and phytochemicals that target key pathophysiological drivers of PCOS, particularly insulin resistance and androgen dysregulation.

### 6.1 Phytochemicals and hormonal regulation in PCOS

Preclinical studies using letrozole-induced PCOS rat models demonstrate that 14-day administration of ethanolic leaf extract of *Parquetina nigrescens* can improve reproductive hormone profiles and associated biochemical parameters (80). The treatment was associated with reduced testosterone levels and increased progesterone and estrogen concentrations, potentially through modulation of aromatase-mediated steroid metabolism. *Foeniculum vulgare* (fennel), a member of the Apiaceae family, contains bioactive compounds including trans-anethole, estragole, and 1,8-cineole, which have been associated with estrogenic, antioxidant, and anti-inflammatory activities (81–84). In a preclinical animal model of PCOS, administration of a chitosan-fennel extract resulted in increased FSH levels, suggesting a possible modulatory effect on reproductive hormone regulation (85).

Evidence from clinical investigations remains limited but indicates potential endocrine effects of certain herbal preparations. A double-blind clinical study evaluating a combination of *Foeniculum vulgare* and *Bunium persicum* in women with PCOS reported reductions in LH levels and hirsutism scores, along with improvements in menstrual cycle characteristics (86). Similarly, *Panax ginseng*, a medicinal plant from the Araliaceae family rich in ginsenosides, has been investigated for its potential influence on endocrine and inflammatory pathways (87). Experimental studies using Korean red ginseng extract reported reductions in circulating testosterone and inflammatory markers such as NF-*κ*B, accompanied by improvements in ovarian morphology and cyst characteristics in preclinical PCOS animal models (88). *Cimicifuga racemosa* (black cohosh) has been investigated for its possible role in modulating HPO axis activity and improving ovulatory function. A randomized controlled trial (RCT) reported that supplementation with *Cimicifuga racemosa* alongside clomiphene citrate during ovulation induction improved cycle outcomes and pregnancy rates in women with PCOS (89). Similarly, *Pimpinella anisum* (anise) contains phytoestrogenic compounds such as anethole, and evidence from preclinical studies in rat models suggests potential effects on menstrual regulation and reproductive hormone balance (83, 84, 90). *Trigonella foenum-graecum* (fenugreek) has received particular attention due to its bioactive constituents, including saponins and diosgenin, and evidence from non-randomized controlled trials and preliminary clinical studies suggests potential improvements in insulin sensitivity, glucose metabolism, and ovarian morphology in women with PCOS (91, 92). Consistent with these findings, evidence from preliminary clinical and experimental studies further suggests that fenugreek extracts may contribute to reductions in ovarian cyst size and improvements in menstrual regularity (93). Collectively, these findings suggest that certain phytochemicals may modulate hormonal pathways involved in PCOS, although further well-designed clinical studies are needed to confirm their therapeutic relevance.

### 6.2 Therapeutic herbs and phytochemicals targeting metabolic dysfunction

A variety of phytochemicals including flavonoids, phenolic acids, terpenoids, and saponins have been investigated for their potential roles in modulating the metabolic disturbances associated with PCOS, particularly insulin resistance, dyslipidaemia, and impaired glucose metabolism, which represent major components of the disorder (94). Evidence from clinical and observational studies has explored the role of herbal formulations in metabolic regulation among women with PCOS, with notable examples including traditional Chinese herbal combinations such as Jia-Wei-Xiao-Yao-San, Gui-Zhi-Fu-Ling Wan, and Dang-Gui Shao-Yao-San, which have been associated with improvements in metabolic parameters and a reduced risk of type 2 diabetes mellitus (T2DM) (95). In addition, several isolated phytochemicals including epigallocatechin-3-gallate (96), curcumin, quercetin, resveratrol (97), and berberine have been evaluated in clinical and experimental studies for their potential effects on insulin resistance and metabolic profiles (Table 1). Previous studies have reported improvements in indices such as the Homeostatic Model Assessment of Insulin Resistance (HOMA-IR) and other metabolic markers in women with PCOS (110, 111). However, the magnitude and consistency of these effects vary across studies.

**Table 1:** Summary of therapeutic phytochemicals explored in the management of polycystic ovary syndrome (PCOS), including their chemical structures, characteristic biological activities, underlying molecular mechanisms, and reported clinical effects on metabolic, endocrine, and reproductive parameters.

**Phytoconstituents**	**Structure**	**Primary Actions**	**Mechanisms of Action and Clinical Impact in PCOS**
Curcumin (phenolic compound)	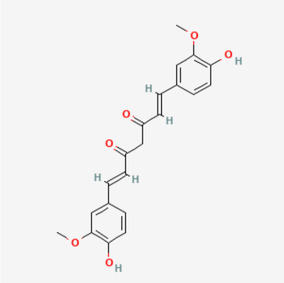	Antioxidant, anti-diabetic, anti-inflammatory properties	Formation of corpus luteum, elevated levels of hormones, antihyperlipidemic, prevention of anovulation (81), decrease in androgen levels and have phytoestrogenic function, reduction of ovarian cysts (98)
Berberine (alkaloid)	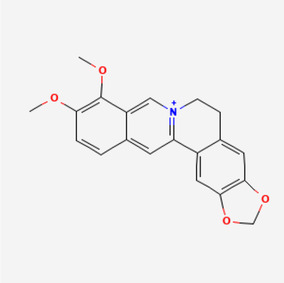	Insulin sensitivity, activate expression of the insulin signalling pathways, inhibits mitogen activated protein kinase pathway	Reduce the levels of hormones, androgens, alleviate hyperlipidaemia, decrease in insulin resistance (99)
Hesperidin (glycoside flavonoid)	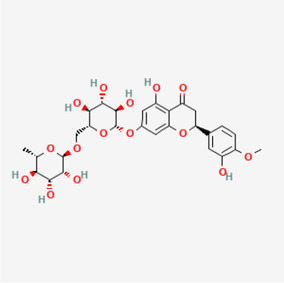	Anti-inflammatory, antimicrobial and antidiabetic effects	Improved follicular growth and development (100), reduce androgen level (101)
Rutin (flavonoid)	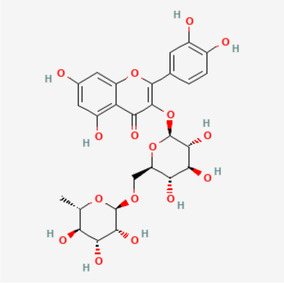	Significant effects on blood glucose level, total protein content, ROS generation and lipid peroxidation	Obesity, irregular estrous cycle, anovulation (98), reduces ovarian dysfunction and systemic insulin resistance
Quercetin (dietary flavonoid)	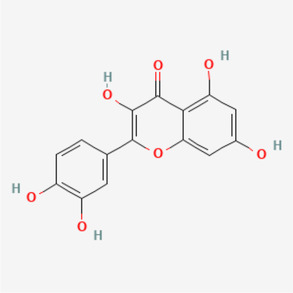	Improved oxidative stress status, anti-inflammatory effects	Improve ovarian disorders, alleviate IR, reduce androgen levels, improve the lipid profile and endothelial dysfunction (102)
Epigallocatechin-3-gallate (green tea catechin/flavonoid)	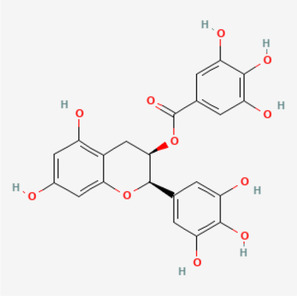	Antioxidant activity, inhibition of pro-inflammatory cytokines (TNF-α, IL-6)	Reduces fasting insulin and glucose levels (alleviating IR), lowers LDL cholesterol, inhibits follicular atresia in the ovaries, and supports weight management by enhancing lipid oxidation (96)
Diosgenin (steroidal sapogenin)	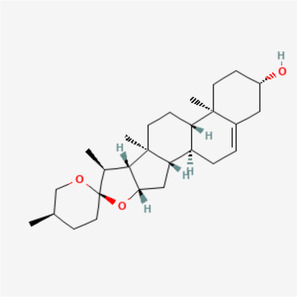	Modulation of the PPAR-γ pathway, reduction of oxidative stress, and inhibition of pro-inflammatory enzymes like COX-2	Enhances insulin sensitivity, regulates lipid metabolism to improve the lipid profile, exerts anti-androgenic effects to restore menstrual regularity, and promotes ovarian follicle maturation (92)
Anethole (phenylpropanoid)	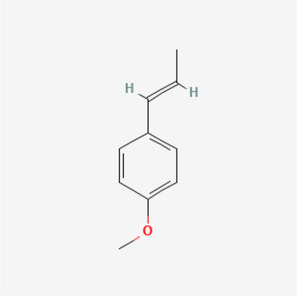	Activation of the AMPK/SIRT1 signaling pathway, antioxidant activity, inhibition of NF-κB-mediated inflammatory responses.	Improves glucose tolerance and insulin sensitivity, reduces hepatic lipid accumulation, exerts estrogen-like effects to help balance the LH/FSH ratio, and mitigates oxidative stress in ovarian tissue (83, 84)
Resveratrol (stilbenoid / polyphenol)	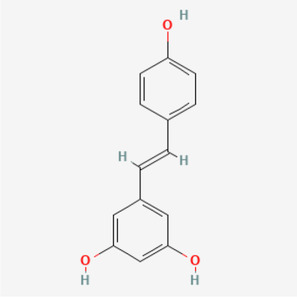	Potent activation of SIRT1 and the AMPK pathway, inhibition of ovarian theca cell proliferation, reduction of proinflammatory cytokines.	Significant reduction in total testosterone and dehydroepiandrosterone sulfate (DHEAS) levels, improvement in insulin sensitivity, and reduction of oxidative stress markers in the follicular fluid to improve oocyte quality (97)
Eugenol (Phenylpropanoid)	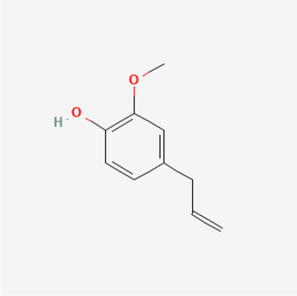	Inhibition of the NF-κB signaling pathway, reduction of oxidative stress via free radical scavenging, and modulation of the PI3K/Akt pathway.	Alleviates insulin resistance by enhancing glucose uptake, reduces systemic low-grade inflammation, exerts anti-androgenic effects by modulating steroidogenesis, and protects ovarian tissue from oxidative damage (103)
Naringenin (Flavanone/Flavonoid)	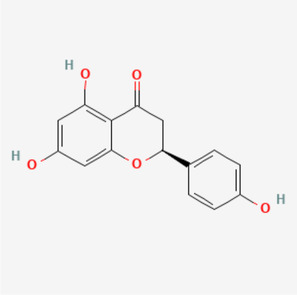	Activation of AMPK and PPAR-γ pathways, inhibition of hepatic gluconeogenesis, and reduction of cytochrome P450 enzyme activity involved in steroidogenesis	Improves insulin sensitivity (alleviating IR), reduces plasma lipid levels (cholesterol and triglycerides), lowers ovarian androgen production, and protects against oxidative damage to follicular cells (104, 105)
Mangiferin (Xanthonoid / C-glycosyl xanthone)	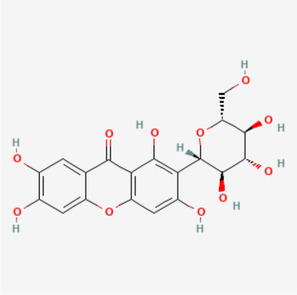	Inhibition of the NF-κB inflammatory cascade, and potent regulation of glucose transporter 4 (GLUT4) translocation	Enhances peripheral insulin sensitivity, significantly reduces serum triglycerides and LDL cholesterol, mitigates oxidative stress in the ovaries, and has been shown to lower hyperandrogenism by modulating steroidogenic enzymes (106, 107)
Apigenin (Flavone / Flavonoid)	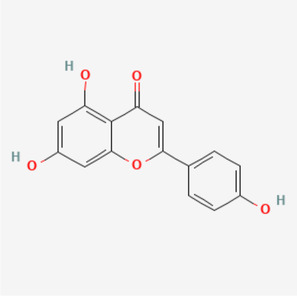	Inhibition of CYP17A1 (the enzyme responsible for androgen synthesis), activation of the PI3K/Akt pathway, and reduction of pro-inflammatory cytokines such as TNF-α.	Directly reduces hyperandrogenism by lowering testosterone production, improves oocyte quality by mitigating oxidative stress in the follicular environment, and enhances insulin sensitivity by modulating glucose metabolism in adipose tissue (108, 109)

Experimental and limited clinical investigations have reported that species such as *Cinnamomum spp.* and *Aloe vera*, which contain polyphenolic and volatile compounds including eugenol, linalool, and cinnamyl alcohol, may contribute to improvements in glycaemic control and lipid metabolism (81, 103, 112). Similarly, extracts derived from *Trigonella foenum-graecum* (fenugreek), particularly preparations containing furostanolic saponins and myo-inositol (Nutricyst-M), have been evaluated in clinical studies where reductions in HOMA-IR and improvements in ovarian morphology were reported (113). Experimental studies further suggest that *Aloe vera* extracts rich in anthraquinones and polyphenols, which may influence folliculogenesis and steroidogenic activity through modulation of lipid and glucose metabolism (87, 114). Herbal species including *Mentha spicata*, *Glycyrrhiza glabra*, and *Linum usitatissimum* have been studied for their possible effects on androgen regulation. Experimental studies in preclinical animal models reported that administration of *Mentha spicata* extracts reduced circulating testosterone levels and improved ovarian morphology in PCOS-induced rats (115, 116). However, these findings are largely derived from preclinical models and require confirmation in well-designed human studies.

A number of individual phytochemicals have also been examined for their potential roles in modulating metabolic and endocrine pathways relevant to PCOS. Compounds such as resveratrol, apigenin, naringenin, catechin, gallic acid, mangiferin, and quercetin have been investigated in experimental models, where they have been associated with improvements in hormonal balance, oxidative stress markers, and inflammatory responses (102, 104–109, 117, 118). Curcumin, a bioactive constituent of *Curcuma longa*, has been evaluated in letrozole-induced PCOS animal models where improvements in glycaemic status, lipid profiles, and inflammatory parameters were observed (119). Similarly, berberine an isoquinoline alkaloid present in medicinal plants such as *Coptis chinensis* has been investigated for its potential metabolic and reproductive effects. Experimental and limited clinical studies suggest that berberine may influence insulin signalling pathways and ovarian function, although these mechanisms remain largely derived from laboratory-based investigations (99, 120, 121). Other phytochemicals such as hesperidin have also been studied within traditional herbal formulations, including Cang-Fu-Dao-Tan Formula, where preclinical and clinical studies suggest possible roles in regulating granulosa cell proliferation and follicular development (100, 122, 123). In addition, plant-derived compounds from species such as *Zanthoxylum armatum* have shown preliminary evidence of influencing androgen-related pathways in experimental models (101). Overall, these findings indicate that several phytochemicals and medicinal plants may influence metabolic and endocrine disturbances associated with PCOS. Nevertheless, much of the current evidence originates from experimental models or small-scale clinical investigations. Variability in herbal preparations, dosage, and study design further limits direct comparisons across studies. Consequently, larger well-controlled clinical trials and standardized phytochemical formulations are required to clarify the potential role of these compounds as adjunct approaches in the long-term management of PCOS.

## 7 Evidence on traditional and complementary approaches in PCOS management

### 7.1 Diet and nutrition

Dietary modification has long been considered an important component of PCOS management in both traditional health practices and contemporary clinical recommendations (124). Historically, dietary patterns emphasizing minimally processed foods, plant-based ingredients, and balanced macronutrient intake have been associated with improved metabolic health. In recent years, several structured dietary approaches including the ketogenic diet, Mediterranean diet, anti-inflammatory diet, and low-glycemic index diet (LGD) have been explored for their potential roles in supporting metabolic regulation in women with PCOS (124, 125). The ketogenic diet, characterized by very low carbohydrate and high fat intake, has been investigated in small clinical studies where improvements in body weight, insulin resistance, and certain metabolic parameters were reported (126). However, evidence remains limited, and concerns regarding long-term adherence, nutritional adequacy, and potential metabolic complications have been raised. Consequently, this dietary strategy is generally considered experimental or short-term and requires careful clinical supervision. Among the dietary patterns evaluated for PCOS, the Mediterranean diet has received relatively greater attention due to its established cardiometabolic benefits in broader populations (127). This dietary pattern emphasizes consumption of fruits, vegetables, whole grains, legumes, nuts, and olive oil, with moderate intake of fish and poultry and limited consumption of red or processed meats (128). Observational studies and small clinical trials suggest that adherence to a Mediterranean-style dietary pattern may improve insulin sensitivity, inflammatory markers, and lipid profiles in women with PCOS, although results remain heterogeneous across studies (129). These benefits are often attributed to its high content of dietary fibre, monounsaturated fats, omega-3 fatty acids, and antioxidant compounds.

Anti-inflammatory dietary patterns, which share several characteristics with the Mediterranean diet, have also been proposed as supportive strategies in PCOS management, based on findings from observational case-control studies suggesting potential benefits in modulating inflammatory and metabolic parameters (130, 131). These approaches prioritize foods rich in antioxidants and anti-inflammatory nutrients such as fatty fish, leafy vegetables, berries, nuts, and whole grains while reducing intake of refined sugars and highly processed foods (132). Although mechanistic links between inflammation and PCOS pathophysiology have been described, clinical evidence directly evaluating anti-inflammatory diets in PCOS remains limited and varies in methodological quality. The LGD represents another nutritional approach that has been investigated in PCOS (133). The LGD emphasizes the consumption of foods that produce smaller postprandial increases in blood glucose, such as whole grains (oats, quinoa, and brown rice), legumes, non-starchy vegetables, and many fruits, thereby helping to improve glycaemic control and insulin sensitivity (134). Several clinical studies suggest that low-glycaemic dietary interventions may contribute to modest improvements in insulin resistance and weight management in PCOS; however, differences in study design, duration, and participant characteristics make direct comparisons challenging. Individualized nutritional counselling from qualified healthcare professionals remains essential to ensure that dietary modifications are safe, nutritionally adequate, and appropriate for each patient's metabolic and reproductive health status. Therefore, dietary strategies should be considered adjunctive approaches that complement, rather than replace, established pharmacological therapies.

### 7.2 Physical activity and movement-based practices

Regular physical activity is widely recommended as an adjunctive strategy in the management of PCOS because of its potential benefits on metabolic health, insulin sensitivity, and body composition (135). Current clinical guidelines generally recommend at least 150 min of moderate-intensity or 75 min of vigorous-intensity exercise per week, along with regular daily movement to support cardiometabolic health (136). Evidence from clinical trials and observational studies indicates that structured exercise interventions may improve insulin resistance, body weight regulation, and cardiovascular fitness in women with PCOS, although study designs and outcomes remain heterogeneous (137). Aerobic exercise, including brisk walking, jogging, cycling, and swimming, is commonly used in PCOS interventions and has been associated with improvements in insulin sensitivity and weight management (135). Recreational activities such as dancing or group-based fitness programs (Zumba) may also contribute to achieving recommended physical activity levels while improving adherence to exercise routines (138). High-intensity interval training (HIIT), characterized by short bursts of vigorous effort (exceeding 90% of maximum heart rate) interspersed with recovery periods, has been investigated in several small clinical studies and may improve aerobic capacity and metabolic parameters compared with continuous moderate-intensity exercise, although long-term benefits and adherence remain uncertain (139). HIIT can be implemented through various exercise formats, including Tabata protocols, circuit training, kickboxing, and functional training programs such as extreme conditioning program training. Resistance or strength training, including body-weight exercises (like squats and push-ups) and light-weight training, has also been explored for its potential to increase lean muscle mass and enhance insulin responsiveness (140, 141). Some observational studies suggest that combined aerobic and resistance training programs may provide greater metabolic benefits than either modality alone, although findings are not entirely consistent.

Movement-based practices with historical or cultural origins, such as yoga, Pilates, and tai chi, have increasingly been investigated for their potential role in PCOS management (142, 143). Evidence from small clinical studies and RCTs suggests that these practices may improve psychological well-being, stress regulation, and certain metabolic or hormonal parameters. Within yoga-based interventions, specific postures (asanas) such as *Baddha Konasana* (Butterfly Pose) is believed to enhance blood flow to the pelvic region and support ovarian function, while *Setu Bandhasana* (Bridge Pose) may stimulate abdominal and pelvic organs and improve circulation to reproductive tissues (144). Similarly, *Bhujangasana* (Cobra Pose) and *Dhanurasana* (Bow Pose) involve gentle compression and stretching of the abdominal region, which may help stimulate ovarian and pancreatic function and improve metabolic activity. In addition, *Surya Namaskar* (Sun Salutation) sequences combine dynamic postures and controlled breathing, which can enhance insulin sensitivity, reduce sympathetic overactivity, and improve overall metabolic health (143, 145–147 ). Despite these promising findings, the current evidence base remains limited by small sample sizes, short intervention durations, and methodological heterogeneity across studies. Therefore, such practices are generally considered adjunctive strategies that may complement, rather than replace, established medical management. Exercise prescriptions for individuals with PCOS should also consider psychosocial factors that influence adherence. Many individuals report body-image concerns and experiences of weight stigma, which may reduce participation in public exercise environments (148). Tailored approaches such as home-based programs, supervised training, or supportive community settings may help improve long-term adherence and engagement with physical activity (149).

### 7.3 Complementary cultural therapies

Several traditional medical systems have historically incorporated holistic approaches for managing menstrual irregularities, metabolic disturbances, and reproductive health concerns that are now associated with PCOS. These practices are rooted in cultural medical frameworks and are increasingly explored in modern research as adjunctive strategies (150). In Ayurveda, PCOS-like conditions are traditionally interpreted as disturbances in the balance of physiological energies (doshas), particularly *Kapha* and *Vata*, and management typically involves individualized dietary recommendations, lifestyle modifications, and herbal formulations (151). Commonly employed botanicals in PCOS management include Shatavari (*Asparagus racemosus*), Ashwagandha (*Withania somnifera*), and turmeric (*Curcuma longa*), which have been frequently investigated in experimental and small clinical studies (119, 152, 153). Shatavari is traditionally valued for its hormone-balancing and antioxidant properties, Ashwagandha functions as an adaptogenic herb that supports stress resilience and endocrine health, while turmeric is widely recognized for its potent anti-inflammatory activity. However, many of these findings originate from preclinical models or small human studies, and the overall clinical evidence remains limited and heterogeneous.

Similarly, Traditional Chinese Medicine (TCM) approaches PCOS-related symptoms through formulations aimed at restoring systemic balance. TCM interventions may involve compound herbal prescriptions or isolated bioactive compounds such as berberine and cryptotanshinone (CRY), which have been evaluated in preclinical experiments and small clinical trials for potential metabolic and endocrine effects (154–156). While some studies report improvements in metabolic markers or ovarian function, methodological variability and limited sample sizes restrict definitive conclusions regarding therapeutic efficacy. Acupuncture, another modality within TCM, has been investigated in several clinical studies for its possible role in regulating menstrual cycles, improving ovulatory function, and influencing metabolic parameters in PCOS (157). Proposed mechanisms include modulation of neuroendocrine signalling and autonomic nervous system activity. Nevertheless, clinical trials have produced mixed findings, and systematic reviews highlight substantial heterogeneity in study design, acupuncture protocols, and outcome measures. Consequently, acupuncture is generally considered a complementary therapy rather than a primary treatment.

The Unani system of medicine adopts a holistic framework in which reproductive disorders are understood as arising from imbalances in the four bodily humors (Akhlat): blood (Dam), phlegm (Balgham), yellow bile (Safra), and black bile (Sauda). Therapeutic interventions are guided by the principle of “therapy by opposites” (Ilaj bil Zid), which aims to restore humoral balance by counteracting pathogenic humors such as Ghair-e-Tabayee Balgham and Sawda (158). Within this framework, therapeutic interventions for PCOS management often involve the use of herbal decoctions (Joshanda), along with agents that promote menstruation (emmenagogues) and support organ function (159). Several botanicals, including *Aloe barbadensis*, *Tephrosia purpurea*, and *Mimosa pudica*, have been explored for their potential therapeutic benefits in PCOS. Among these, *Withania somnifera* (Ashwagandha) and *Tribulus terrestris* (Kharekhask) are frequently regarded as key therapeutic agents, with emerging evidence suggesting that their antioxidant, anxiolytic, immunomodulatory, cardioprotective, and central nervous system (CNS)-modulating properties may collectively contribute to improved management of PCOS-related symptoms (160). Breath-based and meditative practices derived from yoga traditions, including pranayama techniques such as *Kapal Bhati* and *Nadi Shodhana*, have also been explored for their potential role in stress reduction and psychological well-being (161). Given that stress and HPO axis dysregulation may contribute to metabolic and reproductive disturbances in PCOS, these breath-based and meditative practices are sometimes incorporated into integrative management programs (162). However, available studies are typically small and short-term, limiting conclusions about their clinical effectiveness.

Collectively, complementary cultural therapies may offer supportive benefits related to stress management, metabolic regulation, or quality of life, but current evidence is often constrained by small sample sizes, heterogeneous methodologies, and limited high-quality RCTs. Therefore, these approaches are generally considered adjunctive strategies that may complement conventional medical treatments rather than replace evidence-based pharmacological and clinical management of PCOS.

### 7.4 Mind-Body interventions, stress management and psychosocial support

Psychological distress is increasingly recognized as a common comorbidity in women with PCOS (163). Hormonal alterations together with psychosocial stressors including body image concerns, fertility-related anxiety, social stigma, and the financial burden of long-term treatment may contribute to anxiety, depressive symptoms, and reduced quality of life (164, 165). Consequently, psychological support and stress-management strategies are increasingly incorporated into comprehensive PCOS care, as growing evidence from clinical and behavioural studies indicates that these interventions may help alleviate mental health concerns and enhance adherence to lifestyle and medical treatments (166). Particularly, Cognitive-behavioural therapy (CBT) has been investigated in several clinical studies and is commonly recommended in psychological care frameworks for PCOS-related anxiety, depression, and body-image disturbances (156, 161). CBT focuses on identifying and modifying maladaptive thought patterns and behaviours, and some RCTs report improvements in emotional well-being and health-related quality of life (167, 168). Related approaches, such as Acceptance and Commitment Therapy (ACT), which emphasize psychological flexibility and acceptance-based coping strategies, have also been explored in behavioural interventions for chronic health conditions and may offer potential benefits for stress management and lifestyle adherence in PCOS populations (169, 170). However, available research often involves relatively small study populations and varying intervention protocols, which limits generalization of findings.

Mindfulness-based approaches, including meditation-based stress reduction programs and mindfulness-based therapy (MBT), have also been explored as supportive strategies in women with PCOS (156). These interventions aim to improve stress regulation and emotional coping by promoting present-moment awareness and relaxation techniques (171). Related mind-body practices, such as meditation, breathing exercises, and relaxation training, are sometimes incorporated into integrative management programs. These practices may influence stress-related physiological pathways, including hypothalamic-pituitary-adrenal (HPA) axis activity and cortisol regulation (172). A meta-analysis containing twelve RCTs reported improvements in quality-of-life scores and reductions in depressive symptoms among women with PCOS receiving mind-body interventions, although metabolic outcomes were inconsistent (173). A scoping review suggests that meditation and mindfulness-based interventions (MMIs) may have the potential to improve psychosocial well-being and quality of life among women with PCOS; however, the available studies vary considerably in design and intervention protocols (172).

In summary, psychological and mind-body interventions may provide supportive benefits for emotional well-being and stress management in women with PCOS, which can indirectly facilitate adherence to recommended lifestyle and medical treatments. However, these approaches should be viewed as adjunctive components within multidisciplinary care, rather than substitutes for evidence-based pharmacological or clinical management (Table 2).

**Table 2:** Hierarchical clinical framework for the integrative management of PCOS, summarizing first-line, pharmacological, adjunct, and emerging interventions. Approaches are stratified by level of evidence, pathophysiological targets, and clinical role. The framework highlights lifestyle modification as foundational and pharmacological therapy for symptom management, while presenting nutraceuticals, phytotherapy, mind-body interventions, and traditional systems as supportive approaches, acknowledging evidence heterogeneity and the need for further validation.

**Intervention Category**	**Specific Approaches**	**Level of Evidence**	**Clinical Role**	**Primary Targets/Mechanisms**	**Clinical Outcomes/Benefits**	**Key Considerations / Limitations**	**References**
First-Line Interventions (Guideline-Recommended)	Dietary modification (Mediterranean, low-glycemic index, anti-inflammatory, ketogenic diets)	High (RCTs, meta-analyses, international guidelines)	Foundational therapy for all PCOS patients	Insulin resistance, obesity, metabolic dysfunction, ovulatory function	Improved insulin sensitivity, lipid profile, menstrual regularity, ovulation rates, and weight reduction	Requires long-term adherence, variability in patient response, needs individualization	(124, 125)
	Structured physical activity (aerobic, resistance training)	Moderate-High (RCTs, controlled trials, systematic reviews)	Core component of first-line management; adjunct to dietary therapy	Insulin sensitivity, body composition, cardiometabolic health, ovulatory function, psychological well-being	Improved glucose uptake, reduced insulin resistance, enhanced body composition, improved ovulatory function and mental health	Adherence challenges, requires tailoring to individual fitness level and comorbidities, risk of injury with unsupervised or high-intensity exercise	(69, 135–137, 140–143)
	Weight management strategies	High (Guidelines, RCTs, meta-analyses)	Central therapeutic target in overweight/obese PCOS patients	Adiposity, insulin resistance, hormonal imbalance	Modest weight loss (5–10%) associated with improved metabolic parameters, restoration of ovulation, and improved fertility outcomes	Risk of unsupervised dieting, nutrient deficiencies, and weight regain, requires sustainable approach	(67, 68)
	Sleep optimization	Moderate (observational studies, emerging clinical evidence)	Supportive component of lifestyle management	Circadian rhythm, insulin resistance, neuroendocrine regulation	Improved insulin sensitivity, reduced cardiometabolic risk, better hormonal balance and psychological well-being	Limited high-quality interventional data, often under-addressed in clinical practice	(71–75)
Pharmacological Interventions (Core/Standard Care)	Combined oral contraceptives, Insulin sensitizers (metformin), Ovulation induction agents (letrozole, clomiphene), Anti-androgens	High (Clinical Guidelines, large RCTs)	Primary (symptom-specific management)	Hyperandrogenism, menstrual irregularities, infertility, insulin resistance	Regulation of menses, reduced hyperandrogenism, improved fertility outcomes	Potential Adverse effects, contraindications, requires medical supervision	(38–52)
Nutraceuticals & Supplements (Adjunctive)	Inositol (myo-/D-chiro-), Vitamin D, Vitamin E, Omega-3 fatty acids, epigallocatechin-3-gallate, antioxidants, Probiotics	Moderate (RCTs, systematic reviews)	Supportive therapy alongside primary treatment	Modulation of insulin signalling, anti-inflammatory effects, gut microbiota regulation	Improved insulin sensitivity, lipid profile, and hormonal balance	Variability in dosing, formulation, and long-term safety data limited	(53, 54)
Phytotherapy / Herbal Medicine (Adjunctive-Emerging)	*Trigonella foenum-graecum, Foeniculum vulgare, Panax ginseng, Cimicifuga racemosa*, *Rubia cordifolia*, *Parquetina nigrescens, Withania Somnifera, Curcuma longa, Cinnamomum spp.,*	Low-Moderate (small clinical trials, preclinical studies)	Exploratory adjunct, not recommended as standalone therapy	Antioxidant, anti-inflammatory, insulin-sensitizing, endocrine modulation	Potential improvements in glycaemic control, inflammation, and hormonal parameters	Heterogeneous evidence, lack of standardization, unclear dosing, potential herb-drug interactions	(59–62, 77–93, 100, 115, 116, 122, 123)
Physical Activity Subtypes (Adjunct Structuring)	High-intensity interval training (HIIT), Moderate aerobic exercise, Resistance training, Mind-body practices (yoga, Pilates, tai chi)	Moderate-High (clinical trials)	Adjunct optimization of lifestyle therapy	Insulin sensitivity, body composition, stress	Improved insulin sensitivity, body composition, psychological well-being	Suitability varies by patient condition, adherence challenges	(138, 142, 144–147)
Mind-Body & Psychological Interventions (Adjunctive)	Cognitive Behavioural Therapy (CBT), Mindfulness-Based Therapy (MBT), Stress management, Yoga	Moderate (small RCTs, clinical and behavioural studies)	Essential supportive care	Regulation of HPA axis, Anxiety, depression, adherence, quality of life	Improved psychological well-being, quality of life, adherence to therapy	Limited large-scale RCTs, variability in protocols	(156, 161, 166–173)
Complementary Cultural Medical Systems (Adjunctive)	Ayurveda, Traditional Chinese Medicine (TCM), Unani, Acupuncture	Low-Moderate (heterogeneous clinical, preclinical data)	Adjunctive, culturally contextual support	Holistic modulation of metabolic and reproductive function, stress reduction	Symptom relief, improved well-being, potential metabolic benefits	Lack of standardized protocols, variability in quality, limited high-quality RCTs	(57, 58, 150–153, 119, 154–162)
Emerging / Experimental Approaches	Microbiome-targeted therapies, Novel nutraceutical combinations, integrative multi-omics-guided therapies	Low (preclinical, early clinical)	Investigational	Gut microbiota modulation, targeted metabolic and endocrine pathways	Potential future therapeutic benefits	Requires robust clinical validation and safety assessment	(174–176)
Digital & Supportive Care	Online health platforms, mobile health applications, telemedicine, peer-support communities (patient forums)	Low-Moderate (observational studies, behavioural research)	Supportive care enhancement	Enhances patient education, supports self-management, facilitates behavioural change and adherence	Improved disease awareness, engagement, and psychosocial support	Risk of misinformation, requires validation of sources and clinical guidance	(177–179)

## 8 Practical safety considerations for lifestyle and adjunct therapies

As interest in lifestyle modification, nutraceuticals, and complementary therapeutic approaches for PCOS management continues to grow, careful consideration of safety, evidence quality, and clinical applicability becomes increasingly important. Although adjunct strategies including dietary interventions, physical activity programs, phytotherapy, nutraceutical supplementation, and mind-body practices have shown potential benefits for metabolic, reproductive, and psychological outcomes, the supporting evidence remains heterogeneous and is often derived from preclinical studies or small clinical trials (180–182). Herbal products may vary substantially in quality, purity, and concentration of active compounds due to differences in cultivation, processing, and manufacturing standards. In addition, standardized dosing recommendations are lacking for many botanical agents commonly discussed in PCOS management. Potential herb-drug interactions should also be considered, as agents such as berberine and inositol may potentiate the glucose-lowering effects of insulin-sensitizing drugs including metformin, thereby increasing the risk of gastrointestinal intolerance or hypoglycaemia in susceptible individuals (183, 184). Furthermore, modulation of cytochrome P450 enzyme activity by certain herbal compounds may alter the metabolism and efficacy of oral contraceptives and anti-androgen therapies (185). Special caution is warranted for women who are pregnant, planning conception, or undergoing fertility treatments, as several herbal and nutraceutical agents lack robust reproductive safety data and may pose potential risks to fetal development (186). Certain phytoestrogenic herbs and concentrated herbal extracts with hormonal activity may interfere with endocrine balance or embryonic development (187, 188), while supplements including berberine have demonstrated potential uterine-stimulatory or embryotoxic effects in preclinical studies (189, 190). Therefore, their use should be avoided or undertaken only under medical supervision in pregnant women population.

Dietary interventions represent a core component of PCOS management, yet certain dietary strategies also require careful supervision. Although dietary patterns such as the Mediterranean or ketogenic diets influence metabolic health and offer potential health benefits, highly restrictive regimens can also increase the risk of micronutrient deficiencies or unsustainable eating patterns if implemented without appropriate nutritional planning (191). Consequently, individualized dietary guidance from qualified healthcare professionals is recommended, as dietary requirements and therapeutic goals vary considerably among patients (192). Physical activity is a central component of long-term PCOS management, as structured aerobic and resistance training programs may improve metabolic health and body composition, although exercise prescriptions should be tailored to individual health status and comorbidities. High-intensity exercise or unsupervised training programs may not be suitable for individuals with obesity-related joint complications, cardiovascular risk factors, or limited physical conditioning (193, 194). Gradual progression, appropriate exercise selection, and professional supervision may help reduce injury risk and improve long-term adherence. Although experimental and clinical studies suggest potential benefits of traditional therapeutic systems such as Ayurveda, Traditional Chinese Medicine, and Unani medicine, the overall quality of evidence remains heterogeneous across interventions, and standardized treatment protocols are often lacking (195). Consequently, these approaches are generally regarded as supportive strategies that may complement, but should not replace, evidence-based medical therapies. Approaches such as mindfulness-based stress reduction, CBT, meditation, and relaxation practices are increasingly explored within integrative care frameworks (167, 168, 171). However, despite their potential benefits for psychological well-being, the current evidence base remains limited and characterized by heterogeneous methodologies and relatively small clinical samples. In addition, the effectiveness and safety of these interventions may vary depending on implementation, practitioner guidance, and individual patient circumstances.

Digital health platforms and online patient communities have increasingly become important sources of information and peer support for women with PCOS (177–179). Through widely used platforms such as Google and dedicated support websites including https://www.pcosindia.org/, https://www.pcosaa.org/, https://www.mypcosteam.com/ patients are able to access information about disease symptoms, management strategies, and personal experiences shared within online communities. These platforms may enhance disease awareness and provide emotional support by enabling individuals to connect with others facing similar challenges. However, the quality, accuracy, and scientific validity of information available on digital platforms can vary considerably. Users may encounter unverified treatment claims or anecdotal recommendations that are not supported by clinical evidence. Therefore, individuals should be encouraged to critically evaluate the credibility of online sources and verify health information with qualified healthcare professionals before adopting new therapies or lifestyle practices (196, 197). Also, management of PCOS should be individualized, as clinical presentation and therapeutic priorities vary across patient subgroups, including lean vs. obese phenotypes, different age groups, and reproductive or metabolic goals. Overall, the integration of lifestyle strategies, complementary approaches, and psychological support may contribute to long-term PCOS management when implemented within an evidence-based and medically supervised framework. However, these adjunct interventions should be regarded as supportive strategies that complement, rather than replace, established pharmacological and clinical treatments. Careful consideration of safety, quality of evidence, and individual patient characteristics remains essential when incorporating such approaches alongside conventional medical management.

## 9 Future perspectives

Despite growing interest in lifestyle-based and adjunct therapeutic approaches for PCOS, several important knowledge gaps still persists, that warrants further investigation. Future research should prioritize well-designed, adequately powered RCTs to evaluate the efficacy, safety, and long-term sustainability of dietary interventions, structured exercise programs, phytotherapy, nutraceutical supplementation, and mind-body practices in diverse PCOS populations. Standardization of intervention protocols including dosage regimens, formulation quality, and bioactive compound characterization for botanical and nutraceutical preparations remains a critical requirement to improve reproducibility and facilitate meaningful comparison across studies. Greater regulatory oversight and quality control of herbal and supplement products will also be essential to ensure safety and consistency in clinical use. Deeper mechanistic investigations are warranted to elucidate the biological pathways through which adjunct interventions modulate PCOS pathophysiology, particularly with respect to insulin signalling, inflammatory responses, oxidative stress, gut microbiota composition, and neuroendocrine regulation, and to clarify how these interactions contribute to their therapeutic effects. Integration of multi-omics technologies including metabolomics, transcriptomics, and microbiome profiling may help identify novel biomarkers and mechanistic pathways associated with treatment response (174–176).

Future investigations should also consider the substantial heterogeneity of PCOS phenotypes and explore personalized or precision-based approaches that tailor lifestyle and adjunct interventions according to individual metabolic, hormonal, reproductive, and psychosocial profiles. Longitudinal studies evaluating reproductive outcomes, pregnancy safety, and long-term cardiometabolic risk reduction will be particularly important for translating adjunct therapies into clinical practice. In addition, real-world implementation research is needed to better understand patient adherence, feasibility, and accessibility of integrative management strategies across diverse healthcare settings. Digital health technologies, telemedicine platforms, and patient-centered care models may offer opportunities to enhance education, behavioural support, and long-term disease monitoring, although further evaluation of their effectiveness and information reliability is required. Ultimately, the development of multidisciplinary care frameworks that combine evidence-based lifestyle strategies, adjunct therapeutic options, psychological support, and guideline-directed pharmacological treatments may provide a more comprehensive approach to PCOS management. Continued collaboration between clinicians, researchers, and public health professionals will be essential to generate high-quality evidence and translate emerging findings into safe, effective, and individualized therapeutic strategies for women with PCOS.

## 10 Conclusion

Polycystic ovary syndrome (PCOS) is a complex and heterogeneous endocrine disorder characterized by interrelated reproductive, metabolic, and psychological disturbances, necessitating a comprehensive and individualized management approach (198). This narrative review emphasizes the multifactorial nature of PCOS pathophysiology and highlights the importance of integrating evidence-based lifestyle strategies, pharmacological therapies, and carefully evaluated adjunct interventions within a unified clinical framework. Current evidence consistently supports lifestyle modification particularly dietary interventions, physical activity, and weight management as the cornerstone of PCOS management, with pharmacological therapies remaining essential for targeted control of clinical manifestations such as menstrual irregularities, hyperandrogenism, and infertility.

Adjunct approaches, including nutraceuticals, phytotherapy, dietary strategies, physical activity, and mind-body interventions, show potential in modulating the metabolic, inflammatory, and neuroendocrine pathways implicated in PCOS. Nutraceuticals such as inositol, vitamin D, and omega-3 fatty acids have been investigated for their roles in improving insulin sensitivity and hormonal balance (55, 56). In parallel, a range of phytotherapeutic agents including *Rubia cordifolia*, *Parquetina nigrescens*, *Foeniculum vulgare*, *Panax ginseng*, *Trigonella foenum-graecum*, *Bunium persicum* and *Cimicifuga racemosa*, along with other plant-derived bioactives such as berberine, diosgenin, curcumin, and cryptotanshinone have been explored for their potential to modulate key pathophysiological features of PCOS, including insulin resistance and glucose metabolism, hyperandrogenism, ovarian dysfunction, chronic inflammation, and oxidative stress, with evidence derived from experimental and limited clinical studies (89, 91, 92). Dietary interventions remain central within adjunct strategies, with patterns such as the Mediterranean diet, low-glycemic index diet (LGD), anti-inflammatory dietary models, and, in selected contexts, ketogenic approaches demonstrating potential benefits in metabolic regulation and weight management, although their long-term sustainability and adherence vary across individuals (126, 127). Similarly, structured physical activity including aerobic exercise, resistance training, high-intensity interval training, and movement-based practices such as yoga, Pilates, and tai chi has been associated with improvements in insulin sensitivity, body composition, and reproductive function (142, 143).

Complementary cultural approaches derived from traditional systems, including Ayurveda, Unani, and Traditional Chinese Medicine (TCM), incorporate holistic frameworks such as the regulation of physiological energies (*Kapha* and *Vata*), herbal formulations, and practices like acupuncture for effective PCOS management (151). Mind-body interventions, including breath-based practices, meditation, MBT, and CBT, may offer supportive benefits in reducing stress, improving emotional well-being, and enhancing treatment adherence (156, 171). This is particularly relevant given the substantial psychosocial burden associated with PCOS, including anxiety, depression, and body image concerns (199), underscoring the importance of incorporating psychological support into comprehensive care. Despite these promising observations, the supporting evidence across adjunct domains remains heterogeneous and is frequently derived from preclinical studies or small-scale clinical trials with methodological limitations. Accordingly, these strategies should be regarded as supportive measures that complement, rather than replace, established pharmacological treatments, and their integration into clinical practice should be individualized, evidence-informed, and undertaken with careful consideration of safety, quality, and long-term outcomes.

A key contribution of this narrative review lies in synthesizing mechanistic insights linking lifestyle and adjunct interventions with core pathophysiological processes in PCOS, while critically evaluating their translational and clinical relevance. Importantly, the safe integration of these approaches requires careful consideration of product quality, standardization, potential herb-drug interactions, and regulatory oversight to ensure consistency and patient safety in clinical practice (85). Furthermore, PCOS management should be individualized, as clinical presentation and therapeutic priorities vary across patient subgroups, including lean vs. obese phenotypes, different age groups, and reproductive or metabolic goals. Future research should prioritize well-designed, adequately powered clinical trials, standardized intervention protocols, and deeper mechanistic investigations to clarify therapeutic efficacy and long-term reproductive and cardiometabolic outcomes. In parallel, improving patient awareness, addressing psychosocial burden, and incorporating culturally sensitive, patient-centered care models remain essential for optimizing adherence and long-term disease management (200). In conclusion, effective PCOS management requires a balanced, evidence-based, and individualized approach in which lifestyle strategies form the foundation, pharmacological therapies remain central to the management of clinical manifestations, and adjunct interventions are judiciously integrated as supportive measures to enhance metabolic, reproductive, and psychological outcomes without replacing established medical care.

## Glossary

ACT: acceptance and commitment therapy
AMH: anti-mullerian hormone
AMPK: AMP-activated protein kinase
BMI: body mass index
BPA: phthalates and bisphenol-A
CAM: complementary and alternative medicine
CBT: cognitive behavioural therapy
CFDTF: cang-fu-dao-tan
CMA: chlormadinone acetate
CNS: central nervous system
COCs: combined oral contraceptives
CYP19A1: cytochrome P450 family 19 subfamily a member 1
DHEAS: dehydroepiandrosterone sulfate
FAI: free androgen index
FSH: follicle-stimulating hormone
GI: glycemic index
GL: glycemic load
GLUT4: glucose transporter type 4
GnRH: gonadotropin-releasing hormone
HIIT: High-intensity interval training
HOMA-IR: homeostatic model assessment of insulin resistance
HPA axis: hypothalamic-pituitary-adrenal axis
HPO axis: hypothalamic-pituitary-ovarian axis
IBS: intestinal bowel syndrome
IR: insulin resistance
IVF: *in vitro* fertilisation
LGD: Low-glycemic index diet
LH: luteinizing hormone
LHCGR: luteinizing hormone/choriogonadotropin receptor
LPAR3: lysophosphatidic acid receptor 3
MBT: mindfulness-based therapy
MeSH: medical subject headings
MMIs: meditation and mindfulness-based interventions
MUFA: monounsaturated fatty acid
NF-*κ*B: nuclear factor kappa B
OHSS: ovarian hyperstimulation syndrome
OSA: obstructive sleep apnea
PCOS: polycystic ovary syndrome
PI3K: phosphoinositide 3-kinase
PRT: Progressive resistance training
RCTs: randomized controlled trials
REM: rapid eye movement
ROS: reactive oxygen species
SHBG: sex hormone-binding globulin
T2DM: Type 2 diabetes mellitus
TCM: traditional chinese medicine
TCIM: traditional, complementary and integrative medicine
WHO: world health organization
YLDs: years lived with disability
